# An Improved Pigs Weaned per Sow per Year (PSY) Accounting Method Based on Whole-Herd Resource Accounting with Mode-Specific Adaptation for Continuous and Batch Production

**DOI:** 10.3390/ani16213438

**Published:** 2026-10-30

**Authors:** Jinfeng Ma, Qingtian Wei, Peipei Niu, Mengyu Li, Chifeng Chen, Xuemei Xiao, Wangjun Wu, Pinghua Li, Ruihua Huang

**Affiliations:** 1 Sanya Research Institute, Nanjing Agricultural University, Sanya 572025, China; majinfeng@stu.njau.edu.cn (J.M.); lmy2024108@stu.njau.edu.cn (M.L.); michaelchen0414@gmail.com (C.C.); 2026205004@stu.njau.edu.cn (X.X.); wuwangjun2012@njau.edu.cn (W.W.); lipinghua718@njau.edu.cn (P.L.); 2 Key Laboratory of Evaluation and Utilization of Animal and Poultry Resources (Pigs), Ministry of Agriculture and Rural Affairs (Nanjing), Nanjing 210095, China; 3 Institute of Swine Science, Nanjing Agricultural University, Nanjing 210095, China; 4 College of Animal Science and Technology, Nanjing Agricultural University, Nanjing 210095, China; 5 Huai’an Research Institute, Nanjing Agricultural University, Huai’an 223005, China; niupeipei2@126.com; 6 Jiangsu Provincial Strategic Alliance for Technological Innovation of Scale Livestock and Poultry Industry, Huai’an 223005, China; 7 Guangming Agriculture and Animal Husbandry Technology Co., Ltd., Yancheng 224151, China; weiqingtian1130@163.com

**Keywords:** PSY, whole-herd resource accounting, time-weighted average inventory, continuous production, batch production, non-productive days, sow herd management

## Abstract

Pigs weaned per sow per year (PSY) is the core indicator used to evaluate sow-herd reproductive efficiency and a key factor affecting production profitability in commercial pig farms. The traditional PSY calculation uses the average inventory of mated (or parous) sows as the denominator and ignores the resource consumption of non-productive inventory, such as unserved gilts and weaned sows awaiting culling, leading to overestimated reproductive efficiency, concealed costs, and misleading management decisions. The overestimation range of 8–15% has been indicative estimates on non-productive days of commercial sow herds, and the improved method achieved a robustness of ≤3% based on the validation of basic data from pig farming enterprises involved in this paper. In this study, an improved PSY accounting method was developed based on whole-herd resource accounting. Gilts ≥220 days of age and weaned sows not yet culled were incorporated into the basic sow inventory, a dynamic inventory-time-weighting approach was established, and the method was adaptively optimized for continuous and batch production, with mode-specific formulas, validation cases, and an implementation system. It should be noted that traditional PSY and improved whole-herd PSY measure different quantities: the former reflects reproductive efficiency per mated sow, while the latter reflects resource-weighted reproductive efficiency per unit of resource-weighted sow inventory. Case studies on two commercial pig farms showed that the improved method addressed the structural limitations of the traditional formula, produced PSY values of 20–30 piglets per sow per year, and remained robust to parameter variation, with PSY estimates fluctuating by no more than 3% across the feasible range of w (0.60–0.80) in these two cases, and supports refined on-farm cost accounting and standardized cross-farm comparison, although independent multi-farm validation is still required. This study provides a practical tool for reproductive efficiency assessment and refined management in pig farms.

## Simple Summary

Pigs weaned per sow per year is the most widely used indicator of sow-herd productivity and a key determinant of profitability. The traditional calculation uses only mated sows as the denominator, so it ignores the feed, housing, labour and other resources consumed by non-productive animals such as unserved young females and weaned sows that are still kept in the herd. This can overestimate reproductive efficiency by 8–15%, hide production costs, and mislead decisions on culling, replacement-animal development, and facility planning. In this study, we developed an improved calculation method based on whole-herd resource accounting. All females that occupy production resources are included, a time-weighted average inventory is applied, and non-productive animals are weighted according to the resources they consume. The traditional PSY measures reproductive efficiency per mated sow, whereas the improved whole-herd PSY measures resource-weighted reproductive efficiency per unit of herd resources; the two are complementary. Separate formulas were designed for continuous and batch production and validated using real farm records. In the two case farms, the improved method produced PSY estimates that varied by no more than 3% across the feasible range of the weighting coefficient (w = 0.60–0.80), indicating robustness to parameter uncertainty. The method provides a practical, low-cost tool for resource efficiency assessment and cost accounting in commercial sow herds.

## 1. Introduction

Pigs weaned per sow per year (PSY) is the most widely used indicator for benchmarking the reproductive efficiency of sow herds, both between herds within a country and between countries [1]. Over the past three decades, the target values of PSY have increased from approximately 20 to 30 piglets per sow per year as a result of genetic improvement and advances in nutrition and management [1], and PSY remains the primary indicator used in on-farm management decisions, cost accounting and industry benchmarking. In the traditional PSY calculation, the numerator is the total number of piglets weaned during a period, and the denominator is the average inventory of mated females (including mated gilts and sows) during the same period, following the convention adopted in [2,3] of expressing herd productivity as pigs weaned per mated breeding female per year. This denominator covers only females that have entered the reproductive cycle, and it is this scope that defines the conventional PSY as an indicator of reproductive efficiency, that is, piglets weaned per mated sow, rather than an indicator of resource efficiency, which relates output to the total resources occupied by the herd.

As a result, the traditional method reflects the core reproductive efficiency of mated sows while failing to account for the resource consumption of the so-called non-productive inventory, i.e., females that occupy production resources without producing weaned piglets [4,5], such as unserved gilts, weaned sows awaiting culling, sows remaining empty for more than seven days, and aborted sows [6]. It should be clarified that “non-productive inventory” is an accounting category, not a biological judgment: it refers to females that are not contributing weaned piglets during the statistical period concerned, but it does not imply that these animals are biologically incapable of reproduction. A gilt that has not yet been served, a sow in the post-weaning empty period, or a sow that has aborted may all return to the productive cycle subsequently. These categories are included because they consume feed, housing and labour resources without contributing to the weaned-piglet output during the period concerned; excluding them would understate the resource base of the herd. In particular, gilts enter the breeding herd at 220 days of age and continue to occupy resources even if they remain unserved, and weaned sows awaiting culling remain in the herd and consume resources until they are removed. Moreover, as production scale and intensification have increased, the share of non-productive inventory in the whole breeding herd has risen. These non-productive animals contribute to non-productive days (NPD), which reduce the number of possible productive days and therefore limit the number of litters that can be produced per sow per year [7]. When non-productive animals are excluded from the denominator, the traditional PSY reflects the reproductive output per mated sow, but it does not reflect the resources consumed by non-productive inventory. This is not a numerical error in the traditional PSY, but a difference in what the indicator measures. The limitations of the traditional method, which run through resource efficiency evaluation, cost accounting, and turnover management, are summarized in Table 1. The improved whole-herd PSY proposed in this study, by contrast, uses a resource-weighted sow inventory as the denominator and therefore measures resource-weighted reproductive efficiency, i.e., piglets weaned per unit of resource-weighted sow inventory per year. In this sense, the improved whole-herd PSY extends the denominator to all females that occupy production resources and therefore measures a different quantity, resource-weighted reproductive efficiency. The traditional PSY remains valid for benchmarking reproductive efficiency per mated sow, while the improved PSY is intended for resource-use assessment and cost accounting.

These limitations essentially arise from the mismatch between the single accounting logic and the complexity of actual production, which can be analyzed from three dimensions. First, the design of the statistical caliber is limited: the early PSY indicator focused on the core reproductive links and did not fully consider the comprehensiveness of resource occupation in large-scale farms. Ek-Mex [8] used data from 6703 sows on three commercial pig farms and found that the lifetime proportion of non-productive days was 12%. Divided sows into 6 groups according to herd size and found that herd size had a significant effect on PSY: herds with 401–600 sows had the highest PSY, while herds with fewer than 200 or more than 800 sows had the lowest PSY. Angjelovski et al. showed that PSY was 1.31 pigs higher on small farms than on large farms, and that PSY was not correlated with non-productive days (NPD) on small farms, whereas the two were strongly negatively correlated on large farms (r = −0.65) [9]. It is speculated that as farm size increases, the proportion of non-productive inventory may rise, weakening the ability of this indicator to reflect true reproductive efficiency. Second, the adaptation to production modes is insufficient: the popularization of batch production has made the cyclic ‘mating-farrowing’ pattern increasingly prominent, and the traditional continuous-caliber accounting method cannot adapt to the inventory fluctuation pattern of batch production. The two systems differ in their time structure and inventory dynamics. Continuous production is characterized by daily mating, dispersed farrowing and continuous weaning, so the inventory fluctuates smoothly but the weaning output in a given period originates from sows mated approximately 135–142 days earlier. Batch production, by contrast, is organized in fixed batch cycles (e.g., 21 or 28 days), with concentrated mating, farrowing and weaning, so the inventory fluctuates in a stepwise manner and the mating–farrowing–weaning correspondence must be traced batch by batch. If the temporal shift is not applied in continuous production, the weaned piglets in a given period are divided by the inventory of a different cohort of sows, which biases the PSY. Similarly, if batch attribution is not traced in batch production, the mating batch, farrowing batch and weaning batch are misaligned, and the annualization coefficient is applied to the wrong production unit, leading to biased batch PSY and distorted whole-farm aggregation. Bai et al. [10] collected reproductive production data from two large-scale pig farms for three years before and after the transition to batch production, and found that PSY increased significantly after the transition. Duan et al. [11] divided multiparous sows of parities 2–10 into a batch-production group and a traditional continuous-production group, and found that batch production could improve sow reproductive efficiency. However, both studies used the traditional statistical caliber for accounting, without considering the differences in inventory structure between the two modes. The proportion of non-productive sows may be higher under batch production, and the traditional caliber may therefore underestimate the true resource efficiency of batch production. Third, the quantification of resource contribution is lacking: most existing studies proposed the optimization direction of expanding the statistical range but failed to solve the quantification of the contribution of non-productive inventory. Koketsu [6] defined non-productive days (NPD) as the days when mated gilts and sows were neither gestating nor lactating, and further divided NPD into six quantifiable component intervals, thereby providing a methodological basis for evaluating the contribution of non-productive inventory and a methodological reference for the time-weighted averaging used in the present study.

In summary, existing research has clearly identified the core pain points of the traditional PSY accounting, but obvious gaps remain in mode-specific adaptation, quantification of non-productive resources and practical optimization, which provide a clear entry point and research space for the innovation of the present study. Based on the above, this study takes the full coverage of whole-herd resource occupation as its core and constructs a mode-specific improved PSY accounting method. Following the logic chain of ‘citing-evaluating-innovating’, the study absorbs the achievements of existing research and breaks through the technical bottlenecks, providing a complete solution with both theoretical support and operational value for refined management in pig farms.

## 2. Materials and Methods

**Figure 1:**
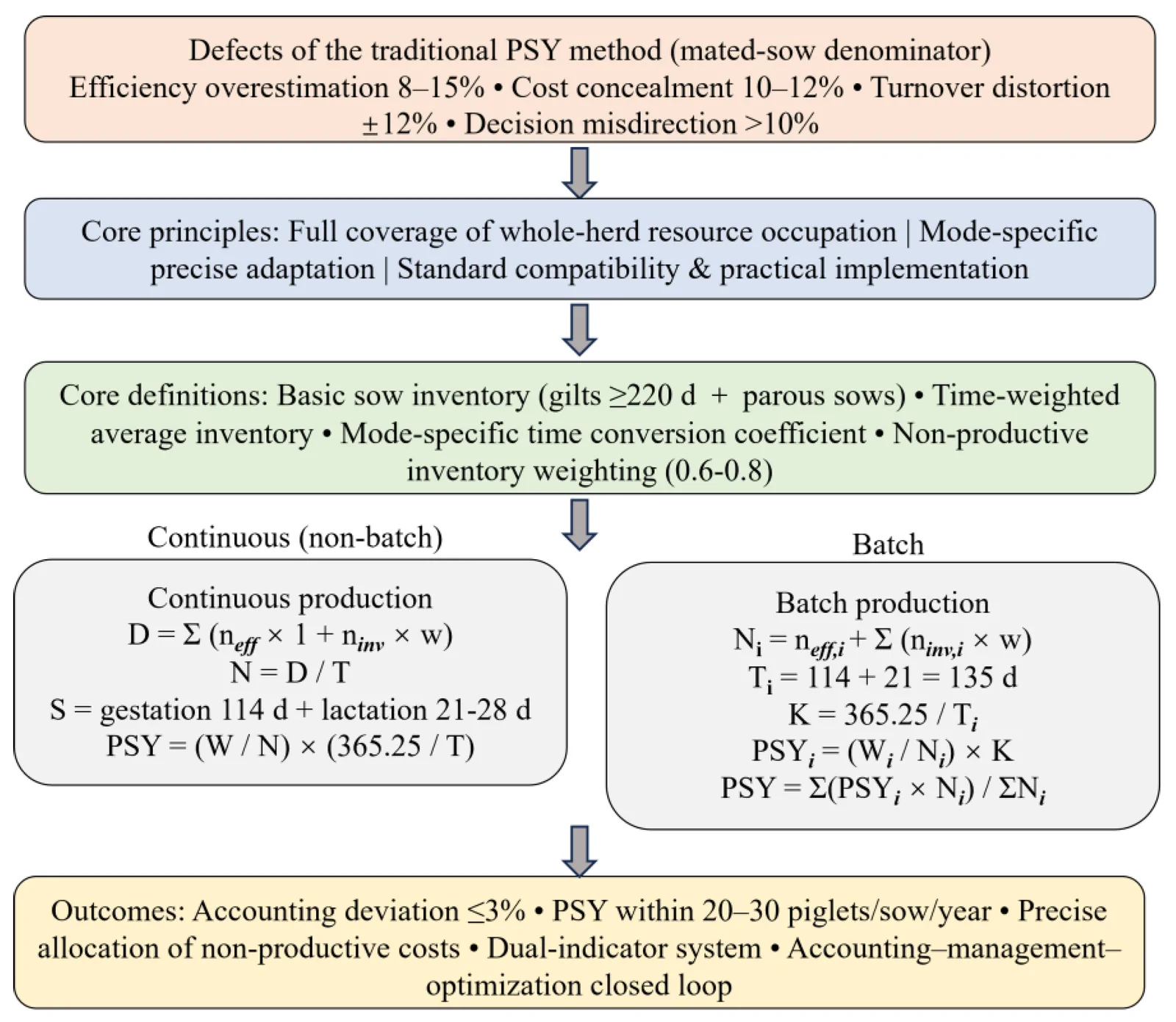
Conceptual framework of the improved PSY accounting system. Note: n*_eff_* is the average daily effective sow inventory, n***_inv_*** is the average daily non-productive inventory, w is the weighting coefficient, N is the time-weighted average inventory, D is the total inventory-days, T is the number of days in the statistical period, S is the shift days (calculated as the average gestation length plus the average lactation length), W is the total number of weaned piglets in the statistical period, N***_i_*** is the batch basic sow inventory, n***_eff_***_,***i***_ is the batch effective inventory, n***_inv,i_*** is the batch non-productive inventory, T***_i_*** is the batch full cycle (the total number of days from mating to weaning for the batch), PSY***_i_*** is the batch PSY, W***_i_*** is the batch number of weaned piglets, and K is the batch annualization coefficient.

To systematically overcome the limitations of the traditional method, the improved method was developed under four guiding orientations—scientificity, practicability, compatibility, and innovation—and a logically coherent accounting system was established, so that the method conforms to academic norms and adapts to production practice. The overall conceptual framework is shown in Figure 1.

### 2.1. Core Principles

Principle of full coverage of whole-herd resource occupation. All females that occupy production resources are included in the statistical scope, so that resource consumption and efficiency evaluation correspond one-to-one. Chen et al. [12] proposed the idea of the cost accounting model of the value flow of pig breeding resources, which provided the core basis for expanding the statistical scope; however, their work lacked a method for quantifying non-productive resources. On this basis, the present study further defines the criteria for non-productive inventory and establishes a dynamic weighting method, effectively solving the main difficulty of quantifying the contribution of non-productive resources.

Principle of mode-specific precise adaptation. According to the rhythm differences between batch and continuous production, differentiated time-conversion and inventory-statistics methods are designed to avoid the bias caused by ‘one-size-fits-all’ accounting. Unlike Duan et al. [11], who compared the differences between production modes, the present study constructs a mode-specific quantitative model, achieving a substantive advance from ‘describing differences’ to ‘solving differences’ and enhancing the practical value of the method.

Principle of standard compatibility and practical implementation. The numerator retains the traditional statistical caliber of ‘total weaned piglets’ to ensure the comparability of data within and across farms, and the denominator adopts the time-weighted average method, which improves accounting accuracy while taking into account data availability. The present study refers to these standards to optimize the annualization conversion coefficients, so that the method is compatible with domestic production practice and internationally comparable, facilitating industrial promotion and application.

### 2.2. Core Definitions

Basic sow inventory. The basic sow inventory is defined as gilts aged 220 days or older (the 220-day threshold is defined as the adaptive breeding-age starting point, with reference to [13]) plus parous sows (including served, gestating, farrowing and empty sows). To clarify the relationship between the 220-day and 250-day thresholds, the eligibility and weighting rules for each age group are defined as follows. Gilts aged 220–249 days are included in the basic sow inventory and are counted as effective inventory with a weight of 1, because they are within the adaptive breeding-age window and are expected to enter the reproductive cycle. From 250 days of age onwards, if a gilt remains unserved, it is reclassified as non-productive inventory and weighted by the coefficient w (0.60–0.80). Thus, the 220-day threshold defines entry into the basic sow inventory, whereas the 250-day threshold defines the point at which an unserved gilt becomes non-productive. Culled individuals within the statistical period are included by time-weighting according to their actual occupation time; deceased individuals are handled under the non-productive inventory rule (the definite date of death minus the mating date, counted in a single lump sum). This rule for culled individuals is newly added in the present study, further improving the accuracy and completeness of inventory statistics. On each day, every animal is assigned to exactly one category; on the day of culling or removal, the animal is counted in its current category for that day only and is excluded from the following day. This inventory therefore represents a resource-equivalent inventory (equivalent gestating sows), rather than a simple head count of mated sows; a non-productive animal with weight w < 1 is counted as consuming less resource equivalent than one gestating sow.

Time-weighted average inventory. Based on the daily (or per-batch) basic sow inventory within the statistical period, the total inventory-days are accumulated according to occupation time. The total inventory-days (D) are calculated as the sum over the statistical period of the daily effective inventory plus the daily non-productive inventory multiplied by the weighting coefficient (Equation (1)), and the time-weighted average inventory (N) is obtained by dividing D by the number of days T in the statistical period (Equation (2)). Annualization is then achieved through the factor 365.25/T applied in the PSY formula (Section 2.3). Compared with the simple arithmetic average inventory method of Koketsu [6], this method introduces a time-weighting factor, effectively solving the accounting bias caused by inventory fluctuation and making up for the lack of a mode-specific design.

D = Σ**_t_** (*n**_eff_*** × 1 + *n**_inv_*** × w) (1)

N = D/T(2)

Mode-specific time conversion coefficient. For continuous production, a ‘reproductive cycle shift coefficient’ is used, defined as the average gestation length plus the average lactation length (S = G + L, with G = 114 d and L = 21–28 d, based on the routine production parameters of the studied farms), which aligns the weaning statistics period with the corresponding mating period and achieves a precise causal match between the numerator and the denominator. The rationale for this shift is that piglets weaned in a given period originate from sows mated approximately G + L days earlier; therefore, the weaning statistics period is shifted back by S days so that the numerator (piglets weaned) and the denominator (sow inventory) refer to the same cohort of sows. For batch production, a ‘batch-cycle annualization coefficient’ is used, defined as 365.25 divided by the number of days of one full batch cycle, so that the per-batch output can be converted to an annual basis.

Non-productive inventory. In the improved denominator, non-productive inventory is incorporated in addition to mated females, and is defined as follows. Non-productive inventory is defined as: (i) gilts of the dominant commercial breeds (Duroc, Landrace, Large White and their crossbred sows) that remain unserved at 250 days of age or older (the threshold may be advanced appropriately for local breeds or breeds containing local-breed bloodlines); and (ii) sows that have been served and remain empty for more than seven days (in cases such as a negative pregnancy check after service, abortion, death, or invalid litters, the invalid production days are calculated as the definite date of occurrence minus the mating date and are included in the non-productive inventory in a single lump sum; after the date of occurrence, animals still in the herd are counted as served sows remaining empty for more than seven days under the normal rule). Where the event occurs before weaning, non-productive days after the event are counted daily from the day following the event until service or culling. Non-productive days are counted consistently across categories: for unserved gilts, from 250 days of age until service or culling; and for served sows remaining empty for more than seven days, from the eighth day after weaning until service or culling. A dynamic weighting coefficient of 0.60–0.80 is assigned to non-productive inventory according to the resource consumption intensity, set within the range observed across non-productive categories in the two case farms. The weighting coefficient is conceptually composed of feed, stall and labour equivalents: w = α·FE + β·SE + γ·LE (α + β + γ = 1), where FE is the feed equivalent (daily feed intake of non-productive animals divided by that of gestating sows), SE is the stall equivalent (≈1.0 where the same stall type is used), and LE is the labour equivalent (≈0.3, based on inspection-time allocation), with the effective (gestating) sow as the reference category (weight = 1). In this study, w was set within the observed range of 0.60–0.80 (baseline 0.70), and the full range was tested in the sensitivity analysis; this weighting thereby enables explicit quantification of the contribution of non-productive resources. The coefficient is a composite resource-use index, not feed or housing alone. The bounds (0.60 and 0.80) are not derived from a formal calculation; they represent the observed range of relative resource consumption across non-productive categories in the two case farms. The same coefficient is applied across categories because these categories share a similar resource-consumption profile (no gestation or lactation); the coefficient is used as a range with sensitivity testing rather than as a category-specific precise value. Hurtgen et al. [14] analyzed the effect of NPD on PSY and provided a theoretical basis for defining non-productive inventory, but did not address its quantification; the present definition, combined with the weighting method, effectively breaks through this technical bottleneck.

### 2.3. Improved Method for Continuous (Non-Batch) Production

The case studies are intended to demonstrate the feasibility of the method, the direction and magnitude of the difference from the traditional method, and robustness to the weighting coefficient; they are not an accuracy validation against an independent gold standard. Continuous production is characterized by continuous mating and dispersed farrowing, with large inventory fluctuations; the core optimization direction is dynamic time weighting and statistical-period shifting to eliminate the hysteresis effect of the reproductive cycle. Baxter et al. [15] focused on inventory management and performance indicator optimization, providing a reference for the data management system of this study, but did not design a calculation formula specifically for continuous production. On this basis, the present study optimizes the accounting logic and adds a weighting coefficient for non-productive inventory, and the shift days are dynamically adjustable to adapt to the lactation-duration differences among farms, thereby improving both accuracy and generality. The calculation formulas are as follows:

The total inventory-days are calculated over a statistical period T of at least 90 days (Equation (1)), which reduces the influence of short-term fluctuations on the accounting result. The time-weighted average inventory is obtained from Equation (2). The shift days (S) are calculated as the average gestation length plus the average lactation length (Equation (3)), and the improved PSY is calculated by Equation (4), in which W is the total number of weaned piglets in the statistical period and N is the time-weighted average inventory of the shifted statistical period.

S = G + L  (G = 114 d; L = 21–28 d)(3)

PSY = (W/N) × (365.25/T)(4)

In Equation (4), N is the time-weighted average inventory of the shifted statistical period, so that the numerator (piglets weaned in the statistical period) and the denominator (sow inventory) refer to the same cohort of sows: the piglets weaned in a given period originate from sows mated approximately S = G + L days earlier. This alignment is verified against the farm’s mating records in the case validation below, which confirm that the sows contributing to the weaning records were mated within the shifted period.

Case validation—continuous production. A medium-sized commercial farm with a continuous-production system was used for validation. The farm uses a single-site design, with a designed sow capacity of 600–650 head, and has been in operation for ~20 years. It covers ~80 mu (≈5.3 ha) and is equipped with automated environmental control and drinking-water systems. Finishing barns use automated feeding lines, whereas breeding barns use manually assisted feeding. Manure handling combines manual dry removal with water-flush removal. Production follows a continuous turnover model, with an annual output of ~15,000 finishing pigs. The statistical period was set from 20 January to 19 April 2025 (T = 90 days), during which 4158 piglets were weaned. Because piglets weaned in this period originated from sows mated approximately S = G + L days earlier, the corresponding shifted statistical period was set from 7 September to 5 December 2024 (S ≈ 135 d, with G = 114 d and L = 21 d for this farm). The mating records of the farm confirmed that the sows contributing to the weaning records in the statistical period were mated within this shifted period. During the shifted period, the average daily effective sow inventory was 628 and the average daily non-productive inventory was 70 (baseline weighting coefficient w = 0.70, the midpoint of the 0.60–0.80 range). The total inventory-days were D = (628 × 1 + 70 × 0.7) × 90 = 60,930 sow-days, and the time-weighted average inventory was N = 60,930 ÷ 90 = 677 sows. The improved PSY was calculated as PSY = (4158 ÷ 677) × (365.25 ÷ 90) ≈ 24.93 piglets per sow per year. Using the traditional method (denominator = average daily inventory of 628 mated sows), PSY = (4158 ÷ 628) × (365.25 ÷ 90) ≈ 26.87, an overestimation of 7.78%, which reflects the difference arising from alternative denominator definitions.

Method advantages. The method precisely adapts to the dynamic inventory characteristics of continuous production; the shift days can be flexibly adjusted; and the weighted quantification of non-productive inventory effectively improves accounting accuracy. In addition, data acquisition relies only on the routine management records of the farm without additional monitoring costs, giving the method good feasibility in small- and medium-sized farms.

### 2.4. Improved Method for Batch Production

Batch production is based on all-in/all-out management with fixed production cycles; the core optimization direction is batch-level time weighting and whole-cycle annualization to correct the logical error of the traditional batch-interval conversion. Previous studies have calculated the inventory and pen occupancy numbers of various pig groups in sow batch production farms [16], which provided a basis for the batch PSY accounting of this study; however, they did not consider weighted averaging or the quantification of non-productive inventory, and the present study makes targeted supplements. The calculation formulas are as follows:

The batch basic sow inventory (N_i_) is the sum of the batch effective inventory and the batch non-productive inventory multiplied by the weighting coefficient. The batch inventory is obtained by first accumulating inventory-days and then normalizing by the batch cycle. The total inventory-days of batch i, D_i_ are calculated as Di = Σ_t_ (n_eff,i,t_ + n_inv,i,t_ × w), where t denotes each day within the batch cycle, n_eff,i,t_ is the effective inventory on day t, n_inv,i,t_ is the non-productive inventory on day t, and w is the weighting coefficient for non-productive inventory (0.60–0.80). The time-weighted average inventory of batch i, N_i_, is then obtained as N_i_ = D_i_/T_i_, where Ti is the batch full cycle (the total number of days from mating to weaning for an individual sow, conventionally 114 + 21 = 135 days, adjustable according to the farm process) (Equation (5)). In the daily summation, each animal is counted only for the days on which it is actually present in the batch. Gilts enter the basic sow inventory on the day they reach 220 days of age. Culled or removed animals are counted up to and including the day of removal, and are excluded from the following day. Deceased animals are counted under the non-productive inventory rule (the definite date of death minus the mating date, multiplied by w and included in n_inv_ in a single lump sum). When an animal changes reproductive state (e.g., from effective to non-productive, or vice versa), it is counted in the new category from the day the change occurs. These rules ensure that each animal is counted in exactly one category on each day, and that no animal is counted more than once; inventory-days are therefore neither omitted nor double-counted across successive batches. The batch weaned piglets (W**_i_**_)_ are the total weaned piglets of the farrowing batch corresponding to the mating batch; the batch full cycle (T_i_) is defined as above; and the batch annualization coefficient (K) is calculated by Equation (6). Two different cycles are used in this method and should not be confused. (a) The 135-day interval (G + L = 114 + 21) is the reproductive cycle of an individual sow; it is used as the denominator of the annualization factor K = 365.25/T_i_ (Equation (6)), so that the per-sow output of one reproductive cycle is converted to an annual basis. (b) The batch cycle (e.g., 21 or 28 days) is the fixed interval between consecutive mating batches; it is used for batch attribution, i.e., to determine which farrowing and weaning batches correspond to a given mating batch, and to determine the number of batches per year. The 135-day cycle does not need to divide evenly into 365 days, because it is not the cycle of the production system; the cycle that repeats throughout the year is the batch cycle. The annualization coefficient is applicable when the batch cycle is fixed and regular; when batch cycles vary within the statistical period, the average cycle length across batches is used as T_i_, so that the annualization remains consistent with the actual production rhythm. The batch PSY (PSY_i_) is calculated by Equation (7), and the whole-farm annual PSY is obtained by the batch-size-weighted average of the batch PSY values (Equation (8)), which reduces the influence of batch-size differences on the whole-farm efficiency evaluation. This weighted aggregation is used because batches differ in size and in the number of sows they contain; weighting by N_i_ ensures that larger batches contribute proportionally to the whole-farm PSY, rather than being averaged equally with smaller batches. When batches differ in duration, the batch weights reflect the inventory contributed by each batch, so that batches of different duration are not treated as if they were of equal length.

N_i_ = D_i_/T_i_, where D_i_ = Σ_t_ (n_eff,i,t_ + n_inv,i,t_ × w) (5)

K = 365.25/T_i_ (6)

PSY_i_ = (W_i_/N_i_) × K (7)

PSY = Σ(PSY_i_ × N_i_)/Σ N_i_ (8)

K converts the per-sow output of one reproductive cycle (T_i_ = 135 days) to an annual basis; because 365.25 is not an integer multiple of 135, K is applied directly rather than rounding the number of cycles per year. The batch cycle (21 or 28 days) governs batch attribution, not the denominator of K.

Key adaptation points. To ensure a logically closed accounting, two core points need to be strictly controlled. (i) Batch attribution tracing: the ‘mating batch–farrowing batch’ and ‘farrowing batch–weaning batch’ correspondence must be strictly traced. These two endpoints must be distinguished: the interval from mating to farrowing is approximately the gestation length G = 114 days, whereas the interval from farrowing to weaning is approximately the lactation length L = 21 days; their sum, G + L = 135 days, is the reproductive cycle of an individual sow, not the interval between batches. Taking a 4-week batch system as an example, a sow mated in batch n is expected to farrow in the batch corresponding to approximately G = 114 days later (114 ÷ 28 ≈ 4.07, i.e., about 4 batch intervals after n), and her piglets are weaned in the batch corresponding to approximately G + L = 135 days later (135 ÷ 28 ≈ 4.82, i.e., about 5 batch intervals after n when rounded up to the next full batch). Accordingly, the farrowing batch and the weaning batch are not necessarily the same batch: the farrowing batch is approximately n + 4, whereas the weaning batch is approximately n + 5. The farrowing and weaning endpoints are therefore treated separately rather than being conflated. This interval should be recalculated when the batch cycle or the gestation–lactation length differs, so that the mating batch, the farrowing batch and the weaning batch always refer to the same cohort of sows. (ii) Dynamic adjustment of non-productive inventory: the non-productive inventory within a batch is weighted according to the actual occupation days, and culled individuals are counted only for the actual inventory days before culling, ensuring the authenticity and accuracy of the batch inventory data. The days between weaning and subsequent service are counted daily in the batch in which the sow is actually present and are not double-counted in the next batch. In addition, when batches overlap or are incomplete, inventory-days are accumulated by actual occupation time rather than by simple addition, so that double counting or omission is avoided.

Worked example (partial-cycle occupancy). Suppose a batch has a cycle of T_i_ = 135 days. One non-productive sow is present in the batch for only 30 days before being culled, with a weighting coefficient w = 0.7; she contributes 30 × 0.7 = 21 sow-days to D_i_. Another non-productive sow is present for the full 135 days, contributing 135 × 0.7 = 94.5 sow-days. If the effective inventory is 140 sows present for the full cycle, it contributes 140 × 135 = 18,900 sow-days. The total is D_i_ = 18,900 + 21 + 94.5 = 19,015.5 sow-days, and the time-weighted average inventory is N_i_ = 19,015.5 ÷ 135 ≈ 140.9 sows. The total 18,900 + 21 + 94.5 = 19,015.5 sow-days can be checked directly: 18,900 from 140 effective sows over the full cycle, 21 from the culled sow over 30 days at w = 0.7, and 94.5 from the full-cycle non-productive sow at w = 0.7. Dividing by T_i_ = 135 gives N_i_ ≈ 140.9. This example illustrates how Equation (5) handles partial-cycle occupancy.

Case validation—batch production. A large-scale farm operating a 3-week batch system was used for validation, with the third mating batch of 2025 taken as the accounting unit. The farm uses a single-site design, with a designed sow capacity of 1200 head, covers approximately 150 mu (≈10 ha), and was built and put into operation in 2013. It is equipped with automated environmental control and fully automated feeding lines, together with intelligent environmental control, automatic drinking-water and precision feeding equipment for breeding stock; manure is handled by a water-flush system. Production follows a 3-week batch-management scheme, with a designed annual output of approximately 28,000 finishing pigs, and the farm has served as the company’s benchmark batch-production farm since its 2020 transition. The accounting batch had 140 effective sows and 17 non-productive sows (baseline weighting coefficient w = 0.70, the midpoint of the 0.60–0.80 range). In this case, all sows were present for the full cycle, so N**_i_** = 140 + 17 × 0.7 = 151.90 sows. With a farrowing rate of 90%, 126 sows farrowed, with an average of 11.5 piglets weaned per litter, so W**_i_** = 126 × 11.5 = 1449 piglets. The batch full cycle was T**_i_** = 135 days and the annualization coefficient was K = 365.25 ÷ 135 ≈ 2.71. The batch PSY was PSY**_i_** = (1449 ÷ 151.9) × 2.71 ≈ 9.539 × 2.71 ≈ 25.85 piglets per sow per year. Using the traditional method (denominator = 140 mated sows), PSY = (1449 ÷ 140) × 2.71 ≈ 10.35 × 2.71 ≈ 28.05, an overestimation of 8.51%. Using the literature method (denominator = 157 sows, i.e., effective plus non-productive sows without weighting), PSY = (1449 ÷ 157) × 2.71 ≈ 9.23 × 2.71 ≈ 25.01, reflecting the effect of not weighting non-productive inventory rather than a difference in accuracy. These results are based on a single batch case; further validation across multiple batches and farms is needed before the method can be generalized.

Method advantages. The method precisely matches the rhythm of batch production and reduces the misalignment problem of the traditional method. The dual optimization of non-productive inventory weighting and batch-level weighted averaging significantly improves the accounting accuracy, and can quickly identify the core problems of low-efficiency batches, providing accurate data support for refined management in large-scale pig farms.

### 2.5. Case Farms and Data Collection

Two commercial farms were used for case validation. Farm A was a medium-sized farm operating continuous (non-batch) production. Daily records of the effective sow inventory, the non-productive inventory (gilts ≥250 days of age, and sows that had been served and remained empty for more than seven days, including cases such as a negative pregnancy check after service, abortion, death, or invalid litters, for which the invalid production days were calculated as the definite date of occurrence minus the mating date and included in a single lump sum; sows returning to estrus or negative on pregnancy check were classified as empty sows under category (ii), consistent with Section 2.2) and the farrowing-house weaning records were extracted from the routine herd management software (e.g., GBS (http://hhpig.foidn.com/ShowProducts.asp?id=142 (accessed on 1 September 2026).), Zhuzhifu) for the statistical periods described in Section 2.3. Farm B was a large-scale farm operating a 3-week batch production system. The batch mating records, batch farrowing performance, tracking of gilts ≥250 days of age and tracking of served sows remaining empty for more than seven days (including the event-based cases described above) were extracted for the third mating batch of 2025 (Section 2.4). Non-productive days for served sows were counted from the eighth day after weaning, consistent with Section 2.2. Unserved gilts aged 220–249 days were counted as effective inventory, and were reclassified as non-productive from 250 days of age, consistent with Section 2.2. The animal categories and non-productive days were defined consistently with Section 2.2. All data were routine production records; no additional monitoring or animal experiments were conducted.

## 3. Results

### 3.1. Validation in the Continuous-Production Farm

In the continuous-production farm, the improved PSY was 24.93 piglets per sow per year, which lies within the reasonable production range of 20–30 piglets per sow per year [1]. The traditional method yielded 26.87, an overestimation of 7.78%, which is consistent with the 8–15% overestimation range identified in Table 1. The inclusion of 70 non-productive females per day (weighted by a coefficient of 0.7) in the denominator reduced the calculated PSY by 1.94 piglets per sow per year. All inputs were extracted from the farm’s routine records: mating dates and parity from the breeding record book, weaning counts from weekly weaning sheets, and daily sow inventory from monthly inventory reports.

Independent corroboration. These case-based estimates are supported by independent published evidence. Across commercial sow herds, non-productive days represent approximately 12.0% of sows’ lifetime reproductive herd life [8]; excluding such a share from the denominator alone would inflate the productivity metric by roughly 13%, which is of the same order as the 8–15% range reported here and is close to the 7.78% overestimation observed in this case. Published estimates further indicate that each non-productive day corresponds to a loss of approximately 0.074 weaned piglets per sow per year [17]. These independently derived magnitudes are consistent with the interpretation that the reported error reduction reflects a real correction of the traditional denominator, although they do not constitute direct validation of the method itself.

### 3.2. Validation in the Batch-Production Farm

In the 3-week batch-production farm, the improved batch PSY was 25.85 piglets per sow per year, also within the reasonable production range. The traditional method overestimated the result by 8.51%, which is near the lower end of the 8–15% range, indicating that the deviation of the traditional method is slightly larger in batch production than in continuous production. The literature method (denominator including non-productive sows without weighting) underestimated the result relative to the improved method, indicating that ignoring the weighting of non-productive inventory leads to an incomplete reflection of resource occupation. Batch-farm inputs were taken from the same record types (mating, weaning and inventory records) and cross-checked against batch-level reports; the accounting unit was the third mating batch.

### 3.3. Multi-Dimensional Comparison of the Three Methods

To further verify the comprehensive advantages of the improved method, a multi-dimensional comparison with the traditional method and the existing literature method was conducted covering accounting accuracy, mode-specific adaptation, cost association and practicality, as shown in Table 2.

### 3.4. Sensitivity Analysis

To assess the robustness of the improved method, the weighting coefficient w was varied from 0.60 to 0.80 in steps of 0.05, while all other parameters were held constant at their case values. In the continuous-production farm, the improved PSY ranged from 25.19 (w = 0.60) to 24.67 (w = 0.80), a change of approximately 2.1%. In the batch-production farm, the improved batch PSY ranged from 26.14 (w = 0.60) to 25.57 (w = 0.80), a change of approximately 2.2% (Table 3). In both cases, the improved PSY remained within the reasonable range of 20–30 piglets per sow per year, and the ranking of the three methods (traditional, literature, improved) did not change.

Sensitivity to other parameters and scenarios. The age thresholds, the seven-day post-weaning interval, the observation period, the production-cycle setting, and scenarios such as herd expansion, concentrated culling, cross-year observation periods and high return-to-estrus rates were not subjected to quantitative sensitivity analysis, because farm-level individual-animal records and longer observation periods were not available. These parameters and scenarios would affect the method mainly through changes in the non-productive inventory and its occupation time. Specifically: Age thresholds: advancing or postponing the gilt age threshold would change the number of gilts classified as non-productive, and thus change the weighted denominator. Seven-day interval: shortening or lengthening the post-weaning interval would shift sows between effective and non-productive inventory. Observation period: extending the statistical period would reduce the influence of short-term fluctuations but would not change the PSY if the numerator and denominator change proportionally. Production-cycle setting: changing the batch cycle would not affect T_i_ or K, because T_i_ is the sow reproductive cycle, not the batch interval; it would only affect batch attribution. Herd expansion: during expansion, the PSY would be temporarily diluted because a larger proportion of the herd would be non-productive. Concentrated culling: concentrated culling would temporarily increase the non-productive share and slightly affect the PSY. Cross-year observation period: a cross-year period would not affect the PSY as long as the annualization factor is correctly applied. High return-to-estrus rate: a higher return-to-estrus rate would increase the non-productive inventory and reduce the PSY.

## 4. Discussion

### 4.1. Reassessment of Reproductive Efficiency Overestimation and Cost Concealment

The case validations showed that the traditional method overestimated PSY by 7.78% in continuous production and 8.51% in batch production, close to or within the 8–15% range identified in Table 1. This overestimation originates from the exclusion of non-productive inventory from the denominator. NPD are recognized as a major cost driver in breeding herds: they reduce the potential number of litters per sow per year, and in a herd weaning 10 piglets per litter, each NPD is equivalent to approximately 0.074 piglets per sow per year [17]. High-performing herds are characterized by fewer NPD across parities [1]. The improved method converts NPD-type inventory into weighted inventory-days so that the feed, housing and labour consumed by non-productive animals are reflected in the denominator. The resulting difference from the traditional method arises from alternative denominator definitions, and is of a magnitude consistent with the independently derived estimates above. It quantifies the share of resource occupation rather than claiming precise cost allocation, and provides a more complete basis for decisions on culling, gilt development and capacity planning, as well as for cost accounting, although the economic effect of the improved method was not quantified in this study and remains to be evaluated with farm-level cost data.

### 4.2. Mode-Specific Adaptation

Continuous production shows large daily inventory fluctuations and a time lag of about 135–142 days between mating and weaning; the reproductive-cycle shift coefficient aligns the numerator (weaning output) with the denominator (mating-period inventory), eliminating the hysteresis bias. In batch production, the inventory fluctuates by batch, and the traditional period-based annualization distorts the causal relationship between the numerator and the denominator; the batch-cycle annualization coefficient (365.25/Ti) and the batch-level weighted averaging eliminate the influence of batch-size differences on the whole-farm evaluation. These tools translate the mode differences described into mode-specific quantitative solutions and address the lack of time-quantification tools. Table 2 shows that the improved method performed best in accounting accuracy. In addition, the improved PSY varied by ≤3% across w = 0.60–0.80 in both modes, indicating robustness to the weighting coefficient.

### 4.3. Quantification of Non-Productive Resources

The weighting coefficient was set within 0.60–0.80 based on the relative resource consumption observed across non-productive categories in the two case farms, and is applied to different categories, including unserved gilts ≥250 days of age, sows empty for more than seven days and aborted sows. The lower and upper bounds (0.60 and 0.80) correspond to the lowest and highest relative resource consumption observed across these categories in the two case farms, and the full range was tested in the sensitivity analysis. Because complete farm-level feed, stall and labour records were not available for all categories, α, β and γ were not estimated by regression, and the formula (Section 2.2) was not used to compute w numerically in this study; it defines the composite structure and the calibration path. Future work should measure FE, SE and LE from farm records and estimate α, β and γ accordingly; as an illustrative initial setting, α:β:γ may be taken as 0.5:0.4:0.1 once these measurements are available. The coefficient is a composite resource-use index (feed + stall + labour), not feed or housing alone. The bounds (0.60 and 0.80) are not derived from a formal calculation; they reflect the observed range in the two case farms. The coefficient has not been independently validated, and its generalisability across breeds, climates and production systems requires further verification. This is a key innovation relative to studies that merely expanded the statistical scope without weighting [12]. The weighting coefficient should be calibrated periodically with farm-specific feed and housing data, and future work should develop adaptive calibration models supported by the Internet of Things and big data. The international productivity benchmarking (e.g., pigs weaned per mated female per year in commercial farms [18]) further confirms the importance of a clearly defined denominator for cross-farm and cross-country comparison. It should be noted that the records used to set the weighting coefficient (feed consumption and stall occupancy records) were collected separately from the data used for method evaluation (inventory and weaning records), so that the coefficient setting does not depend on the evaluation data. In practice, the coefficient should be updated at a fixed frequency, for example quarterly or annually, using farm-specific feed and housing records. To maintain comparability after updates, the updated coefficient is applied only to subsequent statistical periods; historical PSY values are not recalculated unless a retrospective comparison is explicitly required. This ensures that cross-farm and cross-period comparisons remain consistent.

### 4.4. Implementation System

The improved method is supported by a three-component implementation system. First, the data management system: the functional modules of the mainstream herd management software (e.g., GBS, Zhuzhifu) are upgraded to automatically mark gilts ≥220 days of age, dynamically track non-productive inventory and link batch attribution, and a ‘daily check–weekly summary–monthly calibration’ three-level validation mechanism, together with a quarterly comprehensive review, is established to support the accuracy and stability of the accounting data. The present study specifies the functional indicators and implementation path of the software optimization. Second, the statistical standard system: a dual-indicator parallel system is adopted, in which the traditional mated-sow PSY is retained for cross-farm reproductive benchmarking and the whole-herd PSY calculated by the improved method is used for on-farm cost accounting and management optimization, with clear statistical boundaries and application scenarios for the two indicators. The traditional mated-sow PSY measures reproductive efficiency per mated sow, whereas the whole-herd PSY measures resource-weighted reproductive efficiency per unit of resource-weighted sow inventory**.** Standardized statistical workflows are formulated for the two production modes (daily inventory recording–shift calculation–annualization for continuous farms; batch inventory statistics–attribution tracing–weighted averaging for batch farms), and a standardized operation manual is compiled to support consistent results across operators. Third, the technical support system: for gilts ≥220 days of age, routine estrus monitoring and estrus-induction intervention are implemented, and individuals remaining unserved beyond 250 days of age are culled promptly; for weaned sows, estrus monitoring is performed within seven days and hormonal intervention is applied to individuals failing to show estrus within 14 days, so as to minimize NPD [6,19,20,21]. The whole-herd PSY is incorporated into the KPI (key performance indicator) assessment of management personnel, and the stall allocation is optimized according to the production mode (dedicated batch stalls for batch farms; a reasonable gilt-to-sow stall ratio for continuous farms). These measures are proposed to help bridge the practicality gap of previous studies [12] and may facilitate the transformation of the method from a theoretical model to on-farm practice; their implementation effects under a wider range of production conditions remain to be further validated.

### 4.5. Limitations and Future Research

This study is based on case validation in two commercial farms, and the generalizability of the weighting coefficients across breeds, climates and production systems requires further verification. Although the reported error reduction is independently corroborated by published non-productive-day analyses, validation against third-party datasets from additional independent farms, breeds and production systems remains necessary to confirm the transferability of the ≤3% sensitivity (robustness). Future research will focus on three directions: (i) developing a dynamic self-adaptive calibration model for the non-productive inventory weighting coefficient using big data and Internet of Things technologies, so as to realize real-time optimization and precise matching of the coefficient; (ii) conducting multi-scenario validation for different pig breeds and different rearing modes (e.g., fermentation-bed rearing and precision feeding) to refine the mode-specific parameters and expand the applicability of the method; and (iii) conducting long-term multi-farm tracking trials to improve the standardized operation procedure, with the aim of promoting the method as an industry-wide standard for PSY accounting and providing technical support for the high-quality development of the pig industry.

## 5. Conclusions

Focusing on the whole-herd resource accounting and mode-specific adaptation of the PSY indicator, this study constructed an improved PSY accounting method based on ‘dynamic inventory–time weighting’. The method follows the principles of full coverage of whole-herd resource occupation, mode-specific precise adaptation, and standard compatibility with practical implementation; clarifies the core definitions of the basic sow inventory, time-weighted average inventory, mode-specific time conversion coefficient and non-productive inventory; and innovatively introduces a weighted quantification model for non-productive inventory and mode-specific time conversion coefficients, with dedicated calculation formulas and validation schemes for continuous and batch production. In the two case farms, the improved method provided a more complete accounting of whole-herd resource use than the traditional formula, and its PSY estimates remained robust to parameter variation, fluctuating by no more than 3% across the feasible range of w (0.60–0.80), The method therefore supports refined on-farm cost accounting and standardized cross-farm comparison, although independent multi-farm validation against whole-herd resource-accounting records is still required. Further validation across a wider range of farms is needed. Compared with existing research, the method achieves key breakthroughs in non-productive resource quantification, mode-specific adaptation accuracy and the ‘accounting–management’ closed loop, providing a reliable technical tool for accurate reproductive efficiency assessment and refined management in large-scale pig farms, with important practical significance for standardizing the PSY accounting caliber and improving the production efficiency of the pig industry.

## Figures and Tables

**Table 1:** Core limitations of the traditional PSY calculation method and their indicative bias ranges, based on assessments of Chinese commercial sow farms.

Defect Type	Specific Manifestation	Indicative Magnitude of Bias (Not a Formal Calculation; See Note)
Reproductive efficiency overestimation	Gilts ≥220 days of age that remain unserved and weaned sows not yet culled are excluded; the denominator is too small, PSY is inflated and reproductive disorders are concealed	Overestimation 8–15%
Cost concealment	Feed, housing and labour costs of non-productive inventory are not allocated to weaned piglets, leading to underestimation of unit production cost	Underestimation 10–12%
Turnover distortion	The occupation of stalls by non-productive inventory is ignored, biasing stall utilization evaluation and affecting production capacity planning	Utilization deviation ±12%
Management misdirection	The reproductive efficiency of mated sows cannot be distinguished from whole-herd resource efficiency, and the performance of gilt development and post-weaning management is hidden	Decision deviation > 10%

Note: The ranges shown are indicative estimates derived from farm-level assessments on Chinese commercial sow farms in this study; they are not derived from published literature or independent resource-accounting records, they are not universal measured values and may vary with farm size, production system and management level. The 8–15% range is of the same order as the overestimation observed in the two case farms (7.78% and 8.51%) and the published NPD share (approximately 12%); the 10–12%, ±12% and >10% figures are order-of-magnitude estimates for cost concealment, turnover distortion and management misdirection, respectively.

**Table 2:** Multi-dimensional comparison of the traditional, literature and improved PSY accounting methods.

Dimension	Traditional Method	Literature Method	Improved Method (This Study)
Accounting accuracy	Indicative overestimation 8–15%, reproductive efficiency overestimated	Indicative overestimation 4–8%, no non-productive weighting	PSY robust to w (≤3% change across w = 0.60–0.80), weighted quantification of non-productive inventory.
Mode-specific adaptation	No adaptation design; larger deviation in batch production	Preliminary adaptation; no annualization optimization	Mode-specific quantitative model with optimized annualization coefficients
Cost association	Cannot associate non-productive resource costs	Can associate; no cost allocation rules	Improved allocation of non-productive resource costs
Practicality	Easy to operate; lower accuracy	Moderate accuracy; complex operation	Robust to parameter variation in the case farms; compatible with routine data collection

Note: The ratings are indicative estimates based on farm-level assessments and the two case farms in this study; they are not derived from published literature or independent resource-accounting records and should not be interpreted as universally applicable measured values. For the improved method, “≤3% change across w = 0.60–0.80” refers to robustness to the weighting coefficient, as reported in the sensitivity analysis, and is not a measure of accounting accuracy; the accuracy advantage of the improved method is reflected in its weighted quantification of non-productive inventory.

**Table 3:** Sensitivity of the improved PSY to the weighting coefficient w (0.60–0.80).

w	Continuous-Production Farm (PSY)	Batch-Production Farm (PSY)
0.60	25.19	26.14
0.65	25.06	26.00
0.70	24.93	25.85
0.75	24.80	25.71
0.80	24.67	25.57

## Data Availability

Restrictions apply to the availability of these data. The case data were derived from cooperating commercial farms.
